# Revisiting the Immune Trypanolysis Test to Optimise Epidemiological Surveillance and Control of Sleeping Sickness in West Africa

**DOI:** 10.1371/journal.pntd.0000917

**Published:** 2010-12-21

**Authors:** Vincent Jamonneau, Bruno Bucheton, Jacques Kaboré, Hamidou Ilboudo, Oumou Camara, Fabrice Courtin, Philippe Solano, Dramane Kaba, Roger Kambire, Kouakou Lingue, Mamadou Camara, Rudy Baelmans, Veerle Lejon, Philippe Büscher

**Affiliations:** 1 Institut de Recherche pour le Développement (IRD), Unité Mixte de Recherche IRD-CIRAD 177, Montpellier, France; 2 Centre International de Recherche-Développement sur l′Elevage en zones Subhumides (CIRDES), Unité de recherches sur les bases biologiques de la lutte intégrée, Bobo-Dioulasso, Burkina Faso; 3 Programme National de Lutte contre la Trypanosomose Humaine Africaine, Conakry, Guinée; 4 Institut Pierre Richet, Unité de Recherche « Trypanosomoses », Abidjan, Côte d'Ivoire; 5 Programme National de Lutte contre la Trypanosomose Humaine Africaine, Ouagadougou, Burkina Faso; 6 Programme National d'Elimination de la Trypanosomose Humaine Africaine, Abidjan, Côte d'Ivoire; 7 Institute of Tropical Medicine, Department of Parasitology, Antwerp, Belgium; International Centre of Insect Physiology and Ecology, Kenya

## Abstract

**Background:**

Because of its high sensitivity and its ease of use in the field, the card agglutination test for trypanosomiasis (CATT) is widely used for mass screening of sleeping sickness. However, the CATT exhibits false-positive results (i) raising the question of whether CATT-positive subjects who are negative in parasitology are truly exposed to infection and (ii) making it difficult to evaluate whether *Trypanosoma brucei (T.b.) gambiense* is still circulating in areas of low endemicity. The objective of this study was to assess the value of the immune trypanolysis test (TL) in characterising the HAT status of CATT-positive subjects and to monitor HAT elimination in West Africa.

**Methodology/Principal Findings:**

TL was performed on plasma collected from CATT-positive persons identified within medical surveys in several West African HAT foci in Guinea, Côte d'Ivoire and Burkina Faso with diverse epidemiological statuses (active, latent, or historical). All HAT cases were TL+. All subjects living in a nonendemic area were TL−. CATT prevalence was not correlated with HAT prevalence in the study areas, whereas a significant correlation was found using TL.

**Conclusion and Significance:**

TL appears to be a marker for contact with *T.b. gambiense.* TL can be a tool (i) at an individual level to identify nonparasitologically confirmed CATT-positive subjects as well as those who had contact with *T.b. gambiense* and should be followed up, (ii) at a population level to identify priority areas for intervention, and (iii) in the context of HAT elimination to identify areas free of HAT.

## Author Summary

Human African trypanosomiasis (HAT) due to *Trypanosoma brucei (T.b.) gambiense* is usually diagnosed using two sequential steps: first the card agglutination test for trypanosomiasis (CATT) used for serological screening, followed by parasitological methods to confirm the disease. Currently, CATT will continue to be used as a test for mass screening because of its simplicity and high sensitivity; however, its performance as a tool of surveillance in areas where prevalence is low is poor because of its limited specificity. Hence in the context of HAT elimination, there is a crucial need for a better marker of contact with *T.b. gambiense* in humans. We evaluated here an existing highly specific serological tool, the trypanolysis test (TL). We evaluated TL in active, latent and historical HAT foci in Guinea, Côte d'Ivoire and Burkina Faso. We found that TL was a marker for exposure to *T.b. gambiense.* We propose that TL should be used as a surveillance tool to monitor HAT elimination.

## Introduction

Human African trypanosomiasis (HAT) or sleeping sickness is caused by two subspecies of the protozoan flagellate *Trypanosoma brucei*. In West and Central Africa, *T.b. gambiense* causes the chronic form of sleeping sickness, while in East Africa, *T.b. rhodesiense* causes the more fulminant form [1]. *T.b. brucei* is normally not infectious to humans, like other species causing animal African trypanosomiasis (AAT) such as *T. evansi*, *T. congolense*, *T. vivax* and *T. equiperdum*.

After the successful control campaigns dating from 1930 to 1960, *T.b. gambiense* sleeping sickness re-emerged in the 1980s, with tens of thousands of cases treated every year. As a result of control activities, reported cases decreased to a mere 11,382 patients in 2006 [2] and to less than the symbolic number of 10,000 in 2009 [3]. However, along with decreasing incidence, disease control efforts may be discontinued, thus allowing the epidemic to build up again [2]. At present, two West African countries are endemic for HAT [2], [4], [5]. Guinea is the most affected with about 100 HAT cases reported annually from the coastal mangroves. In Côte d'Ivoire, control activities since the 1980s [6] have resulted in a low disease prevalence with a few tens of HAT cases annually, mainly from the Central West foci. In Togo, Ghana, Benin, Mali and Burkina Faso, no autochthonous cases have been reported over the last few years. Although the epidemiological situation remains unknown in several countries, including Liberia and Sierra Leone, HAT elimination in West Africa seems attainable.

Mass screening of the population at risk of *T.b. gambiense* is routinely performed using the card agglutination test for trypanosomiasis (CATT) on select individuals with antibodies against trypanosome antigens. CATT consists of bloodstream form trypomastigotes of *T.b. gambiense* variable antigen type (VAT) LiTat 1.3 purified from infected rat blood, fixed, stained and lyophilised [7]. When a drop of CATT reagent on a plastic card is mixed for 5 min with a drop of blood or diluted plasma or serum, the trypanosomes are agglutinated by antibodies that bind to the surface of the fixed cells resulting in a macroscopic agglutination reaction. Most of these antibodies will react with the VAT-specific epitopes on the cells. These highly immunogenic epitopes are present on the surface-exposed part of the densely packed variant surface glycoproteins (VSG). On living trypanosomes, only these VAT-specific epitopes are accessible for antibody binding. During the production of CATT reagent part of the VSG coat is shed and other epitopes on the VSG molecules that are not strictly VAT-specific, and from other surface proteins embedded between the VSGs, become available for antibody recognition and thus take part in the agglutination reaction [8]. This can lead to false-positive results, compromising the specificity of the test [9].

In the current elimination context in West Africa, when prevalence becomes low or transmission has stopped, the limited specificity of CATT becomes a considerable drawback because it results in low positive predictive values [10]–[12]. Recognising parasitologically unconfirmed but infected CATT-positive cases between many false-positives becomes problematic, since untreated, they may act as a reservoir. Molecular methods such as polymerase chain reaction (PCR), PCR-oligochromatography, NASBA-oligochromatography, real-time PCR and loop-mediated isothermal amplification method (LAMP) have been developed partly to resolve the problem of these unconfirmed CATT-positive subjects, but they also suffer from limited sensitivity, uncertain specificity and poor reproducibility depending on the genome sequence targeted [13]–[15].

In a previous study, the immune trypanolysis test (TL) was shown to be a promising tool to help better understand the phenomenon of nonconfirmed CATT-positive subjects [11]. Trypanosomes are able to change the VAT of their VSG by antigenic variation [16], [17]. During an infection, the host mounts an antibody responses against a variety of VATs [18]. Some VATs are expressed in *T.b. gambiense* (LiTat 1.3, LiTat 1.5 and LiTat 1.6), others in *T.b. rhodesiens*e (ETat 1.2). Detailed studies of trypanosome VAT repertoires have been possible by introducing the TL, which consists of a suspension in complement-rich cavia serum of cloned bloodstream form trypomastigotes, all expressing the same VAT, incubated at 37°C with a test serum. Whenever serum antibodies bind to the VAT-specific epitopes on the trypanosome surface, the cells are lysed through antibody-mediated complement lysis. The TL test is considered 100% specific since in the given test conditions, only VAT-specific antibodies are able to cause lysis of the trypanosomes and such antibodies are absent in noninfected persons [19]. VAT repertoire studies of different trypanosome strains have revealed that some VATs, called predominant VATs, are recognised by almost all *gambiense* sleeping sickness patients, although exceptions do occur. Thus the VAT LiTat 1.3, corresponding to the main CATT antigen, is recognised by all the patients in Côte d'Ivoire while LiTat 1.5, representing a different VSG type, is recognised by almost all Nigerian patients. The combination of VATs LiTat 1.3+ LiTat 1.5+ LiTat 1.6 was able to detect 97% of the patients from eight different countries [19].

The objective of the present study was to evaluate the use of TL with *T.b. gambiense* VATs LiTat 1.3, LiTat 1.5 and LiTat 1.6 aiming at improved epidemiological surveillance of sleeping sickness in West Africa, with special interest in CATT-seropositive persons. TL was performed on plasma collected during medical surveys in several West African HAT foci. Our results argue that TL could be used (i) as a tool to identify CATT-positive subjects who experienced contact with *T.b. gambiense* and (ii) as a surveillance tool to monitor HAT elimination.

## Materials and Methods

### Ethical considerations

All samples were collected within the framework of medical surveys conducted by the national HAT control programmes (NCP) according to the respective national HAT diagnostic procedures. No samples other than those collected for routine screening and diagnostic procedures were collected for the purposes of the present study. All participants were informed of the objective of the study in their own language and signed a written informed consent form. Children less than 12 years old were excluded. For participants between 12 and 18 years of age, informed consent was obtained from their parents. This study is part of a larger project aiming at improving HAT diagnosis for which approval was obtained from WHO (Research Ethics Review Committee) and Institut de Recherche pour le Développement (Comité Consultatif de Déontologie et d'Ethique) ethical committees.

### Study sites

**Figure 1:**
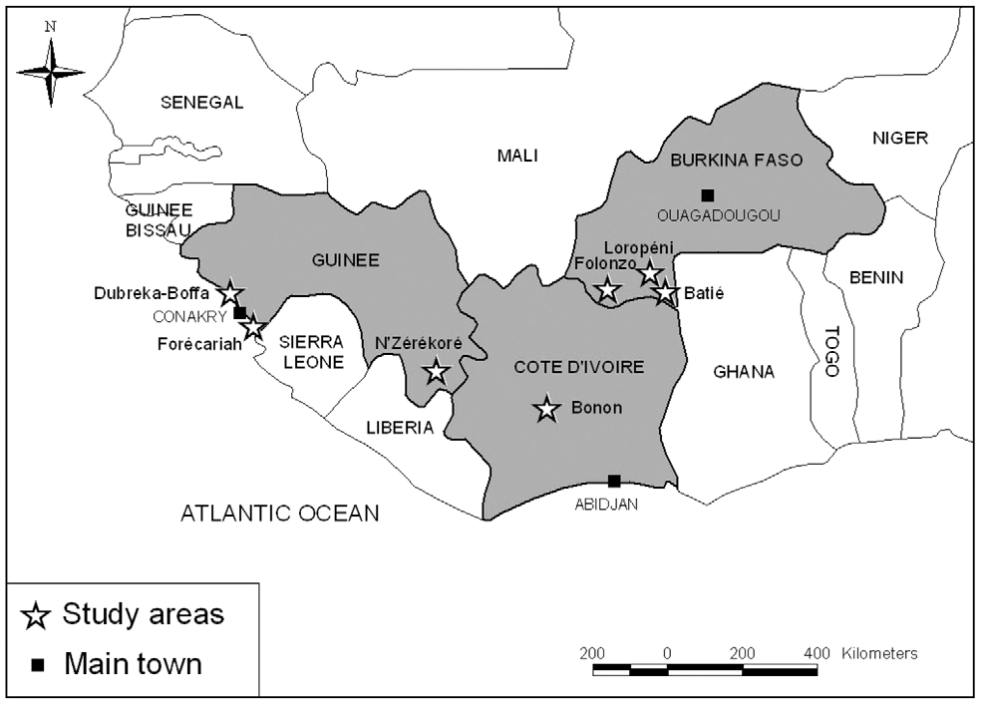
Localisation of sampling areas.

Specimens were collected in three West African countries (Guinea, Côte d'Ivoire and Burkina Faso) in foci with a different epidemiological HAT status (Figure 1).

#### Guinea

The Dubreka/Boffa focus is situated north of Conakry in the coastal mangroves and is currently the most active West African focus with a prevalence of about 1% [20].

The Forécariah focus is situated south of Conakry in the coastal mangroves near Sierra Leone. Sporadic HAT cases reported at the Dubreka treatment centre come from this area.

The N'Zérékoré focus is situated in the woodlands between the savannah and the mesophilic forest near the border with Côte d'Ivoire. It is a historical HAT focus with a risk of re-emergence in the context of socio-political instability and populations moving between Liberia, Côte d'Ivoire and Guinea. Very few recent epidemiological data are available from this area.

#### Côte d'Ivoire

The Bonon focus is situated in the Western central part of the country, between the savannah and the mesophilic forest. Between 1998 and 2003, HAT prevalence in Bonon was about 0.4% [21]. From 2003 onward, HAT prevalence of about 0.1% were observed [22].

#### Burkina Faso

Historical HAT foci of Folonzo, Loropéni and Batié (Fol/Lor/Bat) are located in the South-western part of the country. These areas were recently put under epidemiological surveillance because of a risk of re-emergence of the disease due to the return of agricultural workers from coffee plantations in Côte d'Ivoire where HAT is endemic. Tsetse flies and animal African trypanosomiasis are still present in the area [23], [24], but no HAT cases have been reported over the last few years [25].

### Study subjects

All persons participating in the study were identified during active screening campaigns organised by the NCPs in Guinea, Burkina Faso and Côte d'Ivoire during HAT surveillance activities. Only subjects positive to the CATT/*T.b. gambiense* (CATT-B) performed on blood collected by finger prick and who had never received HAT-specific treatment were included in the study. For CATT-B-positive persons, blood was collected in heparinised tubes and a twofold plasma dilution series in CATT buffer was tested to assess the end titre, i.e. the highest dilution still positive (CATT-P). All persons included in the study underwent parasitological examinations by direct examination of the lymph node aspirate and/or mini-anion exchange centrifugation technique (mAECT) on blood [26]. Thus, three categories of study participants were defined for the purposes of the study:

HAT (patients): CATT-P end titer ≥1/8 and parasitologically confirmed;

SERO (seropositives): CATT-P end titer ≥1/8 but no parasites detected;

SUSP (suspects): CATT-B-positive and CATT-P <1/8 but no parasites detected.

The origin and numbers of participants in each group are detailed in Table 1. Left-over plasma specimens from the subjects were kept at −20°C during field activities, stored at −80°C in the Centre de Recherche Développement sur l′Elevage en zone Subhumide (CIRDES, Bobo-Dioulasso, Burkina-Faso) and sent on dry ice to the Institute of Tropical Medicine (ITM, Antwerpen, Belgium) where TL was performed blindly.

**Table 1:** Study subjects according to the HAT focus and HAT status.

Country	Focus	SUSP	SERO	HAT	Total
Guinea	Dubreka/Boffa	0	17	37	54
Guinea	Forécariah	0	30	28	58
Côte d'Ivoire	Bonon	86	24	6	116
Guinea	N'Zérékoré	0	16	0	16
Burkina Faso	Fol/Lor/Bat	18	25	0	43
**Total**		**104**	**112**	**71**	**287**

Fol/Lor/Bat  =  Folonzo, Loropéni and Batié.

SUSP, SERO and HAT are defined in the Materials and Methods section.

### Trypanolysis test

Cloned populations of *T.b. gambiense* VATs LiTat1.3, LiTat 1.5 and LiTat 1.6 and one *T.b. rhodesiense* VAT ETat 1.2R were used to test plasma as previously described [19]. Briefly, 25 µl of plasma was mixed with an equal volume of guinea pig serum, to which 50 µl of a 10^7^ trypanosomes/ml suspension prepared from infected mouse blood was added. After 90 min of incubation at room temperature, the suspension was examined by microscopy (×250). Trypanolysis was considered positive when more than 50% of the trypanosomes were lysed. ETat 1.2R was a control for the absence of nonspecific trypanolytic activity of the test plasma. Plasma were considered positive (TL+) if positive with at least one of the three variants.

## Results

### CATT-B and CATT-P

A total of 43,373 persons were screened with CATT/*T.b. gambiense* (Table 2). The highest HAT prevalence was observed in Dubreka-Boffa. Low prevalence was observed in Forécariah and Bonon. No patients were detected at the three study sites in Burkina Faso (Fol/Lor/Bat) and N'Zérékoré in Guinea. Seroprevalence of CATT-B and CATT-P end titer 1/8 or higherranged from 0.95 to 3.87% and from 0.31 to 1.21%, respectively, and were not associated with disease prevalence (CATT-B: r^2^ = 0.05, *p* = 0.91; CATT-P end titer ≥1/8: r^2^ = 0.26, *p* = 0.37).

**Table 2:** Results of the medical surveys: sero-prevalence and HAT prevalence.

Study site	Examined population	CATT-B pos		CATT-P pos		HAT	
		number	%	number	%	number	%
Dubreka/Boffa	6795	124	1.82	56	0.82	39	0.57
Forécariah	17571	167	0.95	63	0.36	28	0.16
Bonon	3305	128	3.87	40	1.21	6	0.18
N'Zérékoré	4853	102	2.10	16	0.33	0	0.00
Fol/Lor/Bat	10849	132	1.22	34	0.31	0	0.00
Total	43373	653	1.51	209	0.48	73	0.17

Fol/Lor/Bat  =  Folonzo, Loropéni and Batié; pos  =  positive.

CATT-P pos  =  CATT-P end titer ≥1/8.

### Trypanolysis test

The results of TL on the 287 subjects included in the study are summarised in Table 3. No serum lysed *T.b. rhodesiense* VAT ETat 1.2R, indicating the absence of non-antibody-related trypanolytic factors in the plasma samples.

**Table 3:** TL results according to the study area and status.

Category			SUSP			SERO			HAT		
Study sites		Prev	TL+	TL-	%TL+	TL+	TL-	%TL+	TL+	TL-	%TL+
Dub/Bof	0.57%		0	0	na	15	2	88.2	37	0	100
Forécariah	0.16%		0	0	na	18	12	60	28	0	100
Bonon	0.18%		10	76	11.6	7	17	29.2	6	0	100
N'Zérékoré	0.00%		0	0	na	0	16	0	0	0	na
Fol/Lor/Bat	0.00%		0	18	0	1	24	4	0	0	na
Total			10	94	9.6	41	71	36.6	71	0	100

Dub/Bof  =  Dubreka/Boffa; Fol/Lor/Bat  =  Folonzo, Loropéni and Batié, prev  =  HAT prevalence; na  =  not available; SUSP, SERO and HAT are defined in the Materials and Methods section.

**Figure 2:**
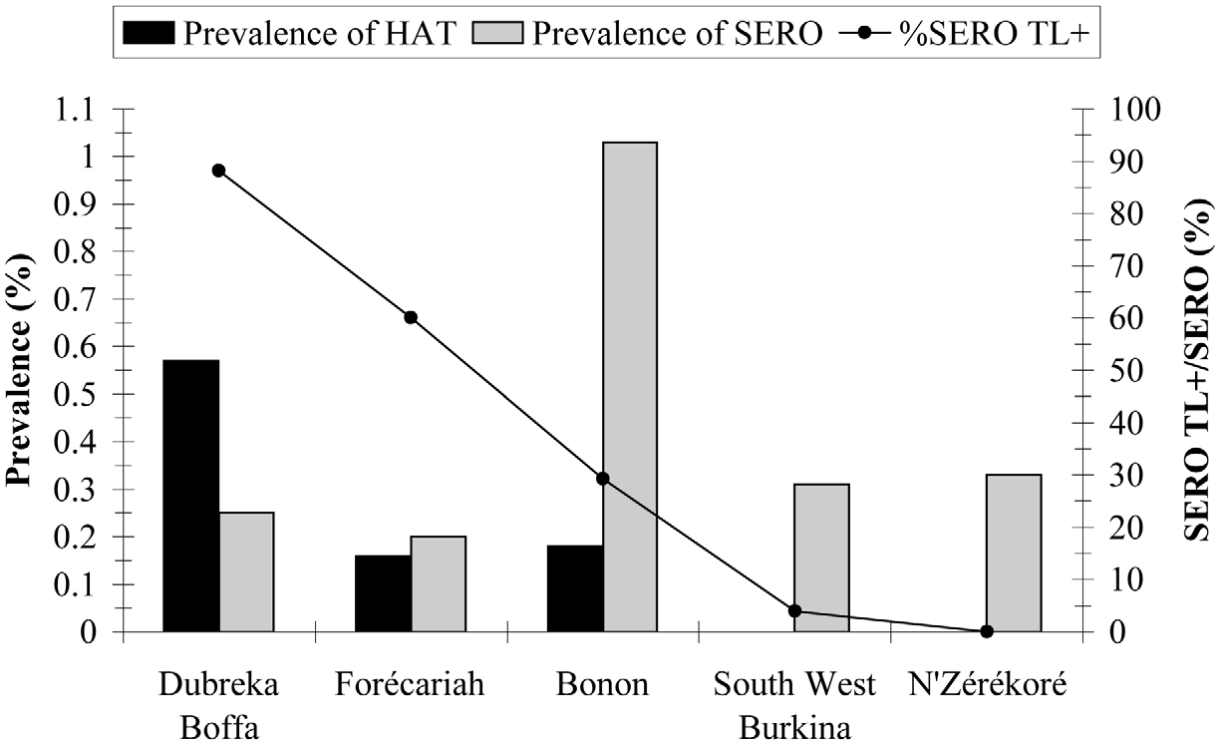
Trypanolysis test is a marker of active HAT transmission. South Western Burkina  =  Folonzo, Loropéni and Batié. The left Y-axis represents the prevalence of HAT (number of HAT cases/examined population) and SERO (number of subjects with CATT-P end titer ≥1/8 but no parasites detected/examined population). The right Y-axis represents the proportion of SERO individuals that were positive to the trypanolysis test.

All 71 HAT patients were TL+. All 18 SUSP from For/Lor/Bat in Burkina Faso were TL− while in Bonon 10 of 86 (11.6%) were TL+. Among the SERO subjects, 41 of 112 (36.6%) were TL+. Interestingly, the percentage of TL+ subjects in the SERO group was correlated with HAT prevalence (r^2^ = 0.84, *p* = 0.03) (Figure 2). It was the highest in the epidemic context of Dubreka/Boffa (15/17, 88.2%), lower in areas of lower HAT prevalence (18/30, 60% and 7/24, 29.2% in Forécariah and Bonon, respectively), whereas no TL+ subjects were detected in areas were HAT was absent, except for one subject in the Fol/Lor/Bat area in Burkina Faso. Table 4 presents the TL results per VAT for TL+ persons by study site. In Bonon, all HAT and SERO subjects were positive for all VATs. Among the ten SUSP, six were also positive for all VATs, whereas four showed different profiles. In Guinea, all 58 of 58 HAT were positive for LiTat 1.3 and LiTat 1.5 and only 38 of 58 were positive in LiTat 1.6. The same trends were observed for SERO: 32 of 33 were positive for LiTat 1.3 and LiTat 1.5 and only 12 of 33 were positive in LiTat 1.6.

**Table 4:** VAT-specific TL-positive profiles according to study site and HAT status.

	LiTat			SUSP	SERO	HAT
Study sites	1.3	1.5	1.6	nb	nb	nb
Dubreka/Boffa	+	+	+	na	10	30
	+	+	−	na	5	7
Forécariah	+	+	+	na	2	15
	+	+	−	na	15	13
	+	−	−	na	1	0
Bonon	+	+	+	6	7	6
	+	+	−	1	0	0
	+	−	+	1	0	0
	+	−	−	1	0	0
	−	−	+	1	0	0
Fol/Lor/Bat	+	+	+	0	1	na

Fol/Lor/Bat  =  Folonzo, Loropéni and Batié; nb  =  number; na  =  not available; SUSP, SERO and HAT are defined in the Materials and Methods section.

## Discussion

This study shows that high prevalence of CATT-positive individuals can be found even in areas were transmission has stopped, presumably owing to false positivity. On the contrary, positivity of TL in SERO subjects was significantly correlated with HAT prevalence and not in nonendemic areas. Thus TL is a useful tool, both to define the epidemiological status of an area when no HAT cases are diagnosed and to improve the monitoring of CATT-positive subjects with no parasitological confirmation, who are currently left out of HAT control strategies in most endemic countries.

### CATT and epidemiological surveillance

The HAT prevalence rates observed in this study are in agreement with recent data on HAT epidemiology in West Africa. Guinea was the most affected country, with 0.57% HAT prevalence in Dubreka-Boffa and 0.16% in the Forécariah focus. No HAT cases were diagnosed in the N'Zérékoré focus. With 0.18% prevalence, HAT is still endemic in the Bonon focus in Côte d'Ivoire. The disease did not re-emerge in the historical foci of Burkina Faso despite the return of agricultural workers from active HAT foci in Côte d'Ivoire since 2002 [25]. In West Africa, areas with disease prevalence approaching zero are becoming common, a recent trend observed in savannah areas [4]. In such areas, CATT-seropositive but parasitologically unconfirmed persons are encountered, making it difficult to evaluate the epidemiological status of the area and to determine what control measures should be applied at both the population and individual levels. This is clearly illustrated by the fact that SERO persons were found in all study sites but their number was not correlated with HAT prevalence (Figure 2). In the historical foci of Burkina Faso and in N'Zérékoré where transmission has stopped, SERO persons may be regarded as false-positives. Aspecific reactions in CATT may have different causes [14] such as cross-reactions with other infectious diseases or transient infections with *T. b. brucei*. Interestingly, the proportion of SERO subjects is highest in Bonon where pig breeding is widespread and where the prevalence of *T.b. brucei* in domestic pigs was reported to be around 70% [27]. Wild fauna and *T.b. brucei* are still present in South-west Burkina Faso [24], where the prevalence of SERO observed in this study is also relatively high. On the other hand, few domestic animals are kept in the Dubreka/Boffa and Forécariah foci in Guinea, where the proportion of SERO is the lowest but which display the highest HAT prevalence. Thus, although CATT is a good serological test for active screening, CATT seropositivity prevalence is not correlated with HAT prevalence, and CATT is not specific enough to evaluate whether *T.b. gambiense* is still circulating in a given area, which is of paramount importance in a disease elimination context.

### Contribution of TL to HAT surveillance

TL was found to be highly sensitive (100% of HAT cases were TL+). Among SUSP and SERO persons, TL+ individuals were only found in areas with proven transmission, except one SERO person in the Fol/Lor/Bat focus in Burkina Faso, which is no longer active. It is noteworthy that this person had worked for 4 years in coffee and cacao plantations in a known HAT focus in Côte d'Ivoire where he may have been exposed to *T.b. gambiense*. Furthermore, a significant correlation was found between the percentage of TL+ SERO persons and the observed HAT prevalence. Our data therefore indicate that TL is a better marker of exposure to *T.b. gambiense* than CATT. The higher specificity of TL observed in this study is explained by the fact that only VAT-specific epitopes can react with antibodies in this test format. Other studies indicated that TL can be more sensitive than CATT since parallel testing with several VATs (LiTat 1.3, 1.5 and 1.6) may reveal infections by *T.b. gambiense* strains not expressing LiTat 1.3, the VAT used for CATT preparation [19], [28]. In addition, TL is based on antibody-mediated complement lysis and can therefore detect much lower antibody concentrations than CATT, which is based on agglutination reactions (unpublished data).

Assuming TL is a marker of exposure to *T.b. gambiense*, the existence in HAT endemic areas of persons harboring *T.b. gambiense*-specific antibodies detectable by TL but without detectable parasites may be explained by one of several hypotheses:

1. These individuals could be patients in an early step of infection with as yet undetectable trypanosomes in blood, lymph or cerebrospinal fluid.
2. They could be asymptomatic carriers with very low parasitaemia. In this study, 53% of the SERO TL+ persons were positive on PCR using the TBR1/2 primers [29] and may actually be infected (data not shown). Asymptomatic infections with undetectable parasitaemia may occur in persons with particular genetic characteristics [12] or be caused by particular *T.b. gambiense* strains inducing silent infections, as demonstrated in mice infected with strains from Côte d'Ivoire [30].
3. They could have experienced a transient episode of *T.b. gambiense* infection, cleared by self-cure or by unreported nonspecific (e.g. autochthonous) treatment. Indeed, in HAT, antibodies may remain present for years after successful cure [10].

Concordant with TL positivity being a marker of contact with *T.b. gambiense* is the fact that lysis profiles to the different LiTat VATs tested were similar in HAT patients and SERO individuals in the different endemic areas. In Côte d'Ivoire, all SERO and HAT patients were positive for all three LiTat VATs. This was not the case in Guinea where only a fraction of HAT patients were positive for LiTat 1.6, as observed in SERO individuals.

### Implication for HAT control strategies

At the individual level, TL can represent a tool for NCPs to identify among CATT-positives those who should be followed up by CATT and parasitological investigations until CATT becomes negative or the person is confirmed as a HAT patient. Whether these seropositives should undergo treatment remains an open question as long as their role in HAT transmission is unknown. We are currently carrying out follow-ups of SERO subjects. Preliminary results indicate that SERO TL+ individuals maintain a strong serological response (CATT and TL) over time, whereas SERO TL− subjects become CATT-negative within several months. Furthermore, HAT patients confirmed during these follow-ups were all from the SERO TL+ cohort (Bucheton, personal communication). In some countries, treatment of unconfirmed persons with CATT-P titers ≥1/16 is already recommended [10]. TL on these persons may avoid unnecessary treatments, as suggested by the nine, five and eight persons in Côte d'Ivoire, Guinea and Burkina Faso, respectively, who had CATT-P titers ≥1/16 but were TL− (data not shown).

**Figure 3:**
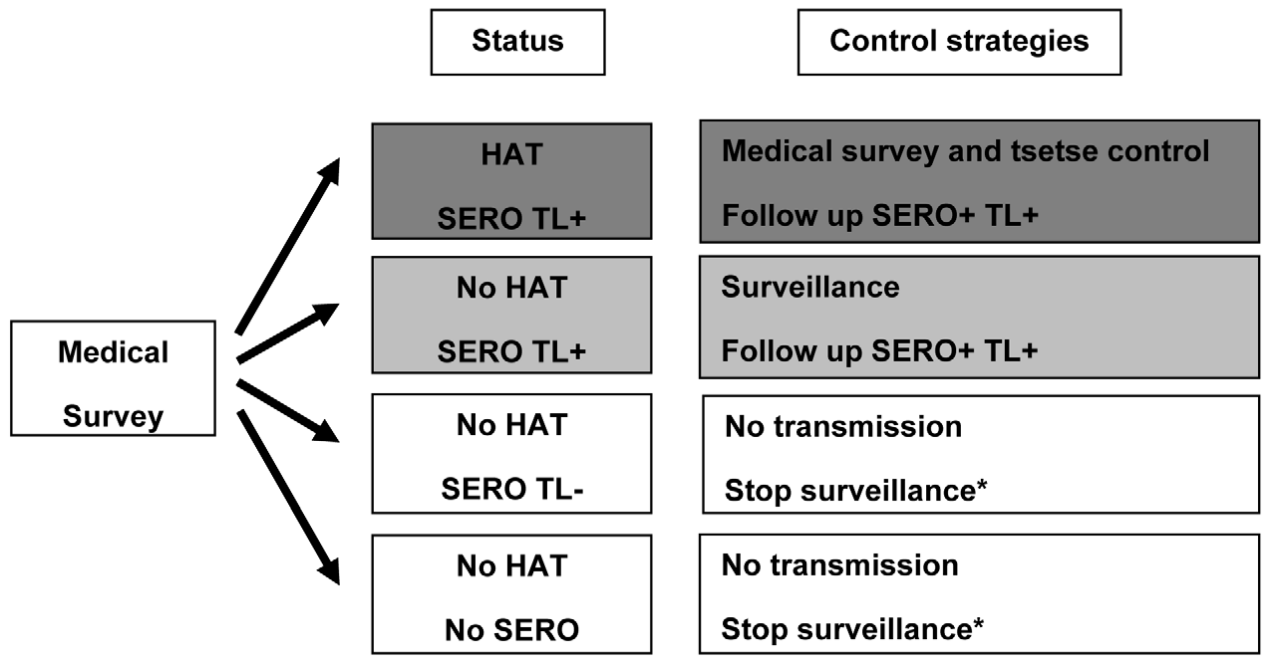
Control strategies: use of trypanolysis test as a marker of *T.b. gambiense* transmission. HAT  =  presence of HAT cases; No HAT  =  absence; SERO  =  presence of subjects with CATT-P end titer ≥1/8 but no parasites detected, No SERO  =  absence; TL+  =  positive in trypanolysis test; TL−  =  negative; * except for a special event, such as population movements, occurs.

At the population level, TL performed on CATT-positive individuals could be a valuable decision tool for NCPs to plan control measures (Figure 3). In active HAT foci, the priority is cutting transmission through active screening and vector control, thus SERO TL+ individuals should be monitored and treated when they become parasitologically positive. Also in areas without HAT, the presence of SERO TL+ cases should sound an alarm, since they indicate contact with *T.b. gambiense*. Continued surveillance of such areas is therefore strongly indicated. In areas without HAT and without SERO TL+ cases, HAT transmission may be considered absent and surveillance may be suspended unless a special event, such as population movement, occurs.

From a practical point of view, the implementation of TL in NCP is hampered by its technological requirements (cryobiology and laboratory animal facilities, availability of VAT-specific control sera, etc.). An alternative test that is applicable in the field and that allows combining several VATs in a single test is the indirect agglutination test LATEX/*T.b. gambiense* [31]. Unfortunately, the purified native VSGs used as antigens in the LATEX/*T.b. gambiense* bear non-VAT-specific epitopes that can lead to false-positive reactions as in the CATT. Investigations to eliminate these non-VAT-specific epitopes in rapid diagnostic tests for HAT are ongoing. In the meantime, adaptation of TL for testing blood collected on filter paper is underway. This would facilitate specimen storage and shipment from the field to the laboratory, as was done for *T. evansi* [32]. Furthermore, as in West Africa almost all TL+ persons are positive in LiTat 1.3, TL with this VAT alone may be sufficient for surveillance purposes in this region. During a WHO meeting held in Bamako in June 2009 with representatives of disease-endemic countries and partners involved in HAT control in West Africa, sleeping sickness control managers welcomed the performance of TL and stated their willingness to collect plasma specimens from SERO cases detected during medical surveys, and send them to CIRDES where TL is now available.

In conclusion, application of the TL test within the framework of medical surveys has provided a better picture of HAT epidemiology in West Africa, thanks to a better characterisation of parasitologically unconfirmed CATT positive subjects. The proportion of TL+ subjects among CATT+ individuals was associated with active HAT foci and can thus be used as a marker for exposure to *T.b. gambiense* even in areas were no HAT cases are currently being diagnosed. TL could thus be regarded as a decision tool for NCPs to decide when surveillance or disease control should be stopped in a given area. The results presented here encourage further investigations, including other endemic countries; into the significance of TL as a tool for improve the knowledge of HAT epidemiology and control.
